# Preparation and characterization of polycaprolactone microspheres by electrospraying

**DOI:** 10.1080/02786826.2016.1234707

**Published:** 2016-09-13

**Authors:** Feng-Lei Zhou, Penny L. Hubbard Cristinacce, Stephen J. Eichhorn, Geoff J. M. Parker

**Affiliations:** ^a^ Centre for Imaging Sciences, The University of Manchester, Manchester, United Kingdom; ^b^ The School of Materials, The University of Manchester, Manchester, United Kingdom; ^c^ School of Psychological Sciences, The University of Manchester, Manchester, United Kingdom; ^d^ College of Engineering, Mathematics and Physical Sciences, University of Exeter, Exeter, United Kingdom; ^e^ Bioxydyn Limited, Rutherford House, Manchester Science Park, Manchester, United Kingdom

**Keywords:** Jian Wang

## Abstract

The ability to reproducibly produce and effectively collect electrosprayed polymeric microspheres with controlled morphology and size in bulk form is challenging. In this study, microparticles were produced by electrospraying polycaprolactone (PCL) of various molecular weights and solution concentrations in chloroform, and by collecting materials on different substrates. The resultant PCL microparticles were characterized by optical and electron microscopy to investigate the effect of molecular weight, solution concentration, applied voltage, working distance, and flow rate on their morphology and size. The work demonstrates the key role of a moderate molecular weight and/or solution concentration in the formation of spherical PCL particles via an electrospraying process. Increasing the applied voltage was found to produce smaller and more uniform PCL microparticles. There was a relatively low increase in the particle average size with an increase in the working distance and flow rate. Four types of substrates were adopted to collect electrosprayed PCL particles: a glass slide, aluminium foil, liquid bath, and copper wire. Unlike 2D bulk structures collected on the other substrates, a 3D tubular structure of microspheres was formed on the copper wire which could find application in the construction of 3D tumor mimics.

## 1. Introduction

Electrospraying and its variant, electrospinning, are two techniques capable of fabricating nano- or micro-sized droplets/fibers from polymer solutions by means of electric forces (Li and Xia 2004; Jaworek 2007). Electrospraying/electrospinning can be used to engineer micro- or nanometer architectures such as particles, fibers, prints/fabrics, or encapsulated particles with controllable nanostructure. Electrospraying/electrospinning parameters (applied voltage, flow rate, working distance, etc.), solution properties (solution viscosity, solution conductivity, surface tension, molecular weight of the polymer, etc.), and environmental conditions (temperature, humidity, etc.) are the main factors that influence the electrospraying/electrospinning process. By changing these factors, polymeric particles/fibers with various morphologies and sizes can be tuned for a specific polymer solution. Compared with the wide range of applications for electrospun fibers (Burger et al. 2006), electrosprayed polymeric particles within the micro- to nanometer range find applications mainly in drug delivery (Bock et al. 2012).

Polycaprolactone (PCL) is suitable for long-term controlled drug delivery due to its high permeability to excipients, excellent biocompatibility, its ability to be fully excreted from the body, and its slow degradation (more than 1 year) (Woodruff and Hutmacher 2010). There have been significant recent studies on the electrospinning of PCL because of the advantages of using this material for tissue scaffolds (Cipitria et al. 2011; Van der Schueren et al. 2011). Compared with poly(lactic-co-glycolic acid) (PLGA), which is the most commonly used polymer in electrospraying (Bock et al. 2012), there have been a relatively limited number of studies on the electrospraying of PCL (Wu and Clark 2007; Enayati et al. 2010; Bock et al. 2011; Guarino et al. 2012). In addition, although the effect of collecting substrates on the physical properties of electrospun nanofibers has been undertaken (Teo and Ramakrishna 2006), no previous reports on the effect of collecting substrates on electrosprayed particle assemblies has been identified. Such information is important for the production of PCL microparticle assemblies with controlled morphology and size. This article focuses on the electrospraying of PCL microparticles onto various collecting substrates. A systematic investigation on the effect of processing parameters, including polymer molecular weight, solution concentration, applied voltage, working distance, and flow rate on the size of PCL particles produced was carried out. A number of different substrates were employed to collect PCL microparticles in order to produce various types of assemblies.

## 2. Experimental section

### 2.1. Materials

Polycaprolactone in three formulations (PCL, number-average molecular weights Mn = 70,000−90,000 (PCL-1), 45,000 (PCL-2), and 10,000 (PCL-3) g mol^−1^) was obtained from Sigma Aldrich (Dorset, UK) and used as received. Chloroform solvent was also purchased from Sigma Aldrich (Dorset, UK).

### 2.2. Preparation of PCL microparticles

**Figure 1.**
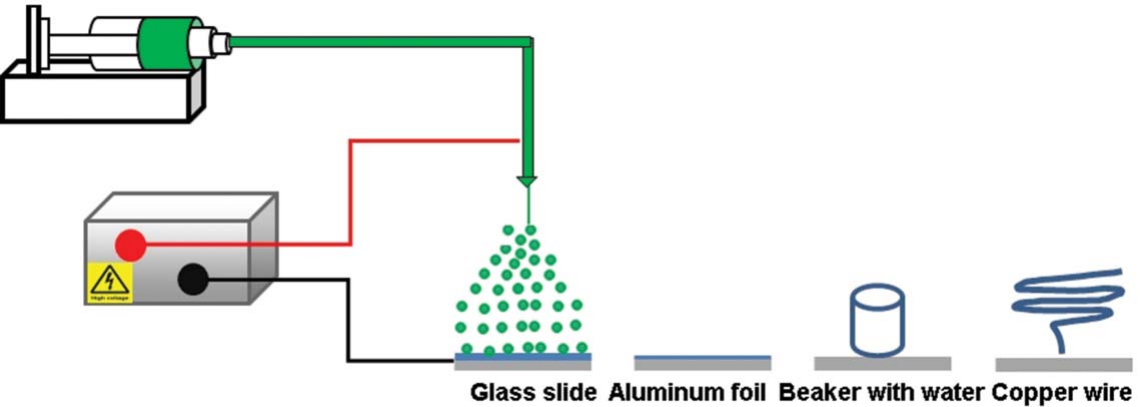
Schematic of the electrospraying process with various collectors (not scaled).

PCL solutions were prepared by dissolving PCL in chloroform and stirring overnight at room temperature. PCL microparticles were fabricated by an electrospraying process (Figure 1) using a setup described previously for electrospinning (Zhou et al. 2011). Briefly, a high-voltage power supply (Glassman High Voltage, Model No. PS/FC30R04.0–22, Hampshire, England) was employed to tune the applied voltage between 0 and 30 kV. A 10 mL plastic syringe with a stainless-steel needle (inner diameter 1.19 mm) mounted on a syringe pump (SP230IWZ, World Precision Instruments, Hertfordshire, England) was used to feed PCL solution to the needle tip with a controllable feed rate. The fabricated particles were then collected on a grounded collector. All experiments were conducted in a fume cupboard under ambient conditions. The influence of a number of parameters on the physical properties of microparticles was studied, including PCL molecular weight, PCL concentration, applied voltage, flow rate, and collector type. For the collector types, a standard microscope glass slide, a flat sheet of aluminium foil, a water bath in a 50 mL beaker at room temperature, and a custom-made spring-shaped copper wire (wire diameter—∼1.2 mm, length—∼100 mm, pitch—∼50 mm, and outer diameter—∼75 mm) were used. All collectors were placed on a grounded aluminium plate (10 cm × 20 cm) located below the spinneret.

### 2.3. Optical and scanning electron imaging

The morphology of the surface of electrosprayed particles was observed using an Olympus BH2-UMA optical microscope and a Philips XL30 FEG SEM or a Phenom G2 pro desktop scanning electron microscope (SEM) with an accelerating voltage of 5 kV. In order to determine the size of the electrosprayed particles, a glass slide was introduced in the setup and held in contact with the collector, in the center of the spraying zone for a few minutes. The electrosprayed PCL particles collected on the aluminium foil were coated with a thin gold film to increase their conductivity before SEM imaging. Image processing software (ImageJ) was used to measure particles' sizes from optical and SEM micrographs.

### 2.4. Statistical analysis

The statistical analysis was performed with the Origin Pro software. A value of *p* < 0.05 was defined as statistically significant. One-way or two-way analysis of variance (ANOVA) followed by a Bonferroni multiple comparison test was used to analyze the particles' size data. One-way ANOVA was used to analyze applied voltage, working distance, or flow rate when solution concentration was fixed. Two-way ANOVA was used to analyze the interaction of solution concentration with applied voltage, or working distance or flow rate on particle size.

## 3. Results and discussion

It is important to stress that the variables involved in the electrospraying process are generally not independent in terms of their impact on particle size. In practice, these variables can be varied only within some finite range to obtain particles of desirable morphology and size and even their relationship with electrosprayed particle sizes holds truly within an optimum range.

### 3.1. Effect of polymer molecular weight

For a polymer solution to be electrosprayed to produce particles, or electrospun for fibers, there are a range of molecular chain entanglement regimes: dilute, semi-dilute unentangled, and semi-dilute entangled. These regimes are determined by the polymer molecular weight and solution concentration. For electrospraying of spherical particles, the regime of choice is the semi-dilute entangled regime, where solid and reproducible particles can be produced (Bock et al. 2012). In terms of the electrospinning of smooth fibers, the concentration of the polymer solution has to be well above the semi-dilute entangled regime (Gupta et al. 2005). However, there have been some recent reports of electrospinning of nanofibers from non-polymeric materials, e.g., cyclodextrin (Celebioglu and Uyar 2011), where no polymer molecular entanglement was present but a high solution concentration/viscosity was still essential.

**Figure 2.**
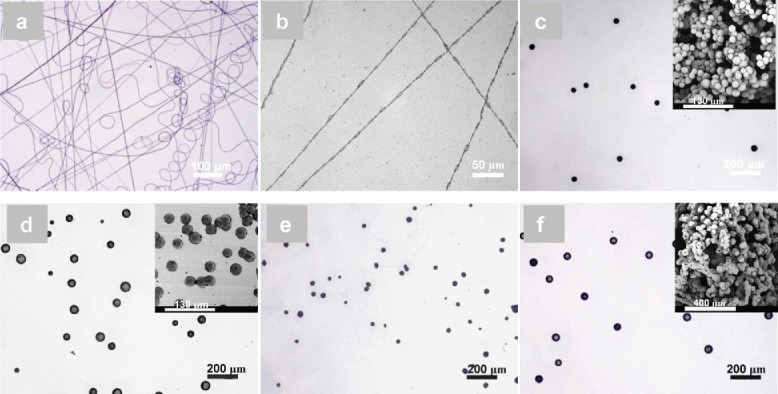
Optical micrographs showing the effects of polymer molecular weight and solution concentration on electrosprayed fibers and particles: (a) smooth fibers from PCL-1, 10 wt.%; (b) beaded fibers from PCL-1, 0.5 wt.%; (c) particles from PCL-2, 9 wt.% (average diameter ± standard deviation: 36.2 ± 2.8 μm, inset: SEM micrograph); (d) particles from PCL-3, 9 wt.% (47.2 ± 15.7 μm, inset: SEM micrograph); (e) particles with fibrils from PCL-2, 11 wt.%; (f) particles from PCL-2, 30 wt.% (50.3 ± 5.1 μm, inset: SEM micrograph).

Solution PCL-1 was first electrosprayed since it used a molecular weight that has been previously reported for the electrospinning of fibers (Cipitria et al. 2011). As shown in Figures 2a and b, it was not possible to produce spherical particles from PCL-1 by electrospraying, even when reducing the solution concentration from 10 wt.%, where smooth PCL fibers were produced, to 0.5 wt.%, from which beaded PCL fibers were made. PCL particles were readily produced from a 9 wt.% PCL solution when the molecular weight was decreased to 45k (PCL-2) and 10k (PCL-3); particles were produced with average diameters of 36.2 ± 2.8 μm and 47.2 ± 15.7 μm, respectively (Figures 2c and d). When the concentration of PCL-2 was slightly increased from 9 wt.% to 11 wt.%, a number of very fine PCL fibers were produced, along with microparticles (Figure 2e). However, for PCL-3, particles with an average diameter of 50.3 ± 5.1 μm were produced, even from a 30 wt.% solution (Figure 2f), indicating a low molecular weight of PCL could produce spherical particles from a wider range of solution concentrations. The effect of PCL molecular weight and solution concentration on the formation of particles is in agreement with similar findings for the electrospraying of PLGA (Meng et al. 2009). SEM imaging (inset in Figure 2c) further revealed that the electrosprayed PCL particles from PCL-2 had a spherical shape and a smooth surface. For PCL-3, the particles appeared to collapse when the PCL concentration was 9 wt.% (inset in Figure 2d). A hole with an average size of approximately 8.4 ± 2.3 μm was however formed on each particle surface when the PCL concentration was increased to 30 wt.% (inset in Figure 2f). It has been previously reported that the surface morphology of PCL particles changes from a smooth surface to a rough corrugated one when the solution concentration is reduced and that a lower molecular weight leads to the formation of particles with a hole in the surface (Xie et al. 2006). A solvent evaporation-induced phase separation was proposed to explain the formation of holes in electrosprayed PCL particles (Wu and Clark 2007).

**Figure 3.**
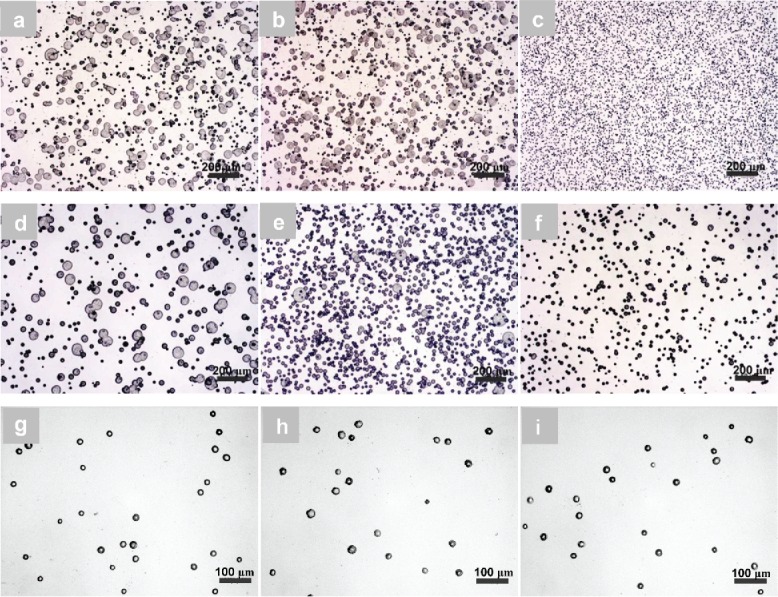
Effect of applied voltage on the morphology and size of PCL particles electrosprayed from PCL-2. (a) 1 wt.%−6.5 kV; (b) 1 wt.%−7.5 kV; (c) 1 wt.%−8.5 kV; (d) 5 wt.%−6.5 kV; (e) 5 wt.%−7.5 kV; (f) 5 wt.%−8.5 kV; (g) 7 wt.%−8 kV; (h) 7 wt.%−9 kV; (i) 7 wt.%−10 kV.

**Figure 4.**
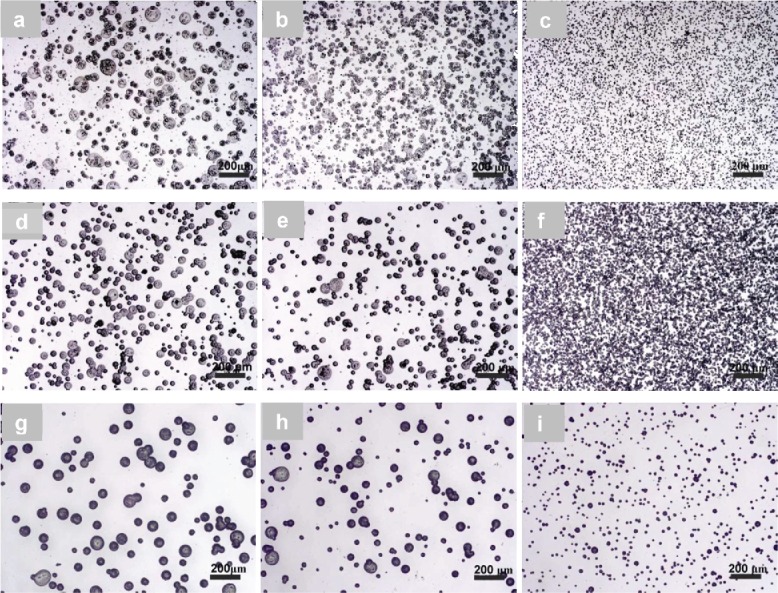
Effect of applied voltage on the morphology and size of PCL particles electrosprayed from PCL-3 solution. (a) 1 wt.%−6.5 kV; (b) 1 wt.%−7.5 kV; (c) 1 wt.%−8.5 kV; (d) 5 wt.%−6.5 kV; (e) 5 wt.%−7.5 kV; (f) 5 wt.%−8.5 kV; (g) 9 wt.%−9 kV; (h) 9 wt.%−10 kV; (i) 9 wt.%−11 kV.

### 3.2. Effect of applied voltage

A sufficient applied voltage is required to overcome the surface tension of the droplet at the needle tip and thus controls the morphology and size of electrosprayed particles. It is generally accepted that an increase in the applied voltage can lead to a decrease in the size of microparticles. However, very high voltages cannot be used because of the onset of an unstable jet. There is usually a lower and an upper voltage limit, which allows a stable jet in electrospraying, resulting in monodispersed particles and better repeatability and reproducibility.

For PCL-2 with 1 wt.% and 5 wt.% concentrations, an increase in the applied voltage tended to produce more uniform and smaller PCL particles (Figures 3a–f). For example, in the case of 1 wt.% PCL-2 solution, the average diameter of the particles significantly increased (one-way ANOVA, *p* < 0.05) from 16.9 ± 5.9 μm at 6.5 kV to 18.6 ± 7.6 μm at 7.5 kV, and then dramatically decreased to 10.0 ± 1.6 μm at 8.5 kV (Table 1). There were also significant changes in particle diameters for a 5 wt.% PCL solution; from 27.6 ± 11.4 μm through 23.8 ± 6.8 μm, to 21.9 ± 5.6 μm (Figures 3d–f) with an increasing applied voltage. These dramatic reductions in diameter are likely to be because the stronger electric field at the higher voltage forces the conical meniscus at the needle tip to deform and to break into smaller droplets. As shown in Figures 3g–i, for a 7 wt.% PCL solution, the change in PCL particle sizes became much less noticeable than those from 1 wt.% and 5 wt.% solutions. Bonferroni-corrected *post hoc* tests further pointed toward no significant difference (*p* > 0.05) in the mean sizes of particles produced from a 7 wt.% PCL-2 solution when the applied voltage was changed from 9 kV to 10 kV, although there was a significant change from 8 kV to 9 kV. This indicated that the effect of the applied voltage could become weak with a relatively high concentration of the solution. It should be noted that too high a voltage resulted in an unstable spray (data not shown). Two-way ANOVA tests further revealed that the solution concentration and applied voltage had a significant interaction (*p* < 0.05). As shown by the histograms in Table 1, PCL particles produced using 1 wt.% and 5 wt.% solutions had wider size distributions than those generated from a 7 wt.% solution; for all three PCL solutions, a higher applied voltage could benefit the formation of uniform spherical particles.

**Table 1.** Mean and standard deviation of diameters and distributions of PCL-2 particles produced under different applied voltages.

Applied voltage *d_n_* (µm) Solution concentration	6.5 kV	7.5 kV	8.5 kV	Particle diameter distributions
1 wt.%	16.9 ± 5.9 μm	18.6 ± 7.6 μm	10.0 ± 1.6 μm	
5 wt.%	27.6 ± 11.4 μm	23.8 ± 6.8 μm	21.9 ± 5.6 μm	
7 wt.%^*^	18.7 ± 2.4 μm	21.1 ± 3.0 μm	19.3 ± 2.6 μm	

* For the 7 wt.% PCL solutions, the applied voltage was 8 kV, 9 kV, and 10 kV. The relationship between sphere sizes and applied voltage/solution concentration was also plotted using *x-y-z* spatial coordinates. These plots were included as supplementary material.

For PCL-3 with concentrations of 1 wt.% and 5 wt.%, higher applied voltages also produced more uniform (as shown by the size distributions in Table 2) and spherical particles with smaller diameters (Figures 4a–c). For example, there were significant decreases in the particle diameter from 27.0 ± 9.9 μm to 10.4 ± 2.1 μm and from 31.2 ± 13.8 μm to 13.9 ± 4.7 μm (Figures 4d–f), respectively, when the applied voltage was varied from 6.5 kV to 8.5 kV. When the solution concentration was increased to 9 wt.%, it is also noted that when the applied voltage was increased, a dramatic decrease in the particle diameter occurred (from 53.4 ± 17.6 μm to 18.7 ± 5.3 μm) (Figures 4g–i). This observation is consistent with previous findings. However, Bonferroni *post hoc* tests showed no significant changes in mean particle diameter (from 31.2 ± 13.8 μm to 30.0 ± 12.7 μm) occurred at a 5 wt.% concentration when the voltage was changed from 6.5 kV to 7.5 kV. Two-way ANOVA showed there is a significant relationship between solution concentration and applied voltage (*p* < 0.05). Table 2 shows the size distributions of particles from the solutions with different concentrations. Clearly, PCL-3 particles produced at a lower applied voltage have broader size distributions and larger average sizes than those formed at higher applied voltages, especially for PCL solutions with 1 wt.% and 5 wt.% concentrations.

**Table 2.** Mean and standard deviations of PCL-3 particle diameters and their distributions, produced under different applied voltages.

Applied voltage *d_n_* (µm) Solution concentration	6.5 kV	7.5 kV	8.5 kV	Particles diameter distributions
1 wt.%	27.0 ± 9.9 μm	18.9 ± 7.0 μm	10.4 ± 2.1 μm	
5 wt.%	31.2 ± 13.8 μm	30.0 ± 12.7 μm	13.9 ± 4.7 μm	
9 wt.%^*^	53.4 ± 17.6 μm	39.0 ± 18.0 μm	18.7 ± 5.3 μm	

* For the 9 wt.% PCL solution, the applied voltage was 9 kV, 10 kV, and 11 kV. The relationship between sphere sizes and applied voltage/solution concentration was also plotted using *x-y-z* spatial coordinates. These plots were included as supplementary material.

From these results it can be seen that a relatively higher applied voltage was required to produce PCL particles with uniform size, particularly for low concentration solutions of low molecular weight PCL. However, it is worth mentioning that for a given solution concentration, the range of applied voltage for a stable electrospraying process was typically narrow and was mainly influenced by flow rate. In the electrospraying process, applied voltage may affect several factors including the electrostatic force on the polymer solution droplet, polymer mass fed out from a tip of needle, etc. A balance between these factors may determine the final morphology and size of electrosprayed particles.

### 3.3. Effect of working distance

The working distance in the electrospraying process is the distance from the needle tip of the spinneret to the grounded collector. This distance determines the time that the solvent can take to evaporate when an electrosprayed droplet accelerates toward the grounded collector. An increase in the working distance reduces the electric field strength but increases the travel time of the droplets, which has an effect on the evaporation of the solvent. Therefore, it could have a dual effect on the resultant electrosprayed particles, up to a point at which a particular factor may become dominant. It was noted that PCL particles tended to merge with neighboring particles due to the presence of residual solvent when the working distance was too short (for example <10 cm). However, a working distance greater than 30 cm could lead to a significant loss of particles to the surroundings as they travel to the nearest grounded object in order to discharge. Therefore, similar to previous studies on the electrospraying of polystyrene (Park and Lee 2009) and chitosan (Arya et al. 2009), the working distance had to be optimized to ensure the sufficient evaporation of any remaining solvent in the PCL particles, thus alleviating their merging, and appropriate collection efficiency.

**Figure 5.**
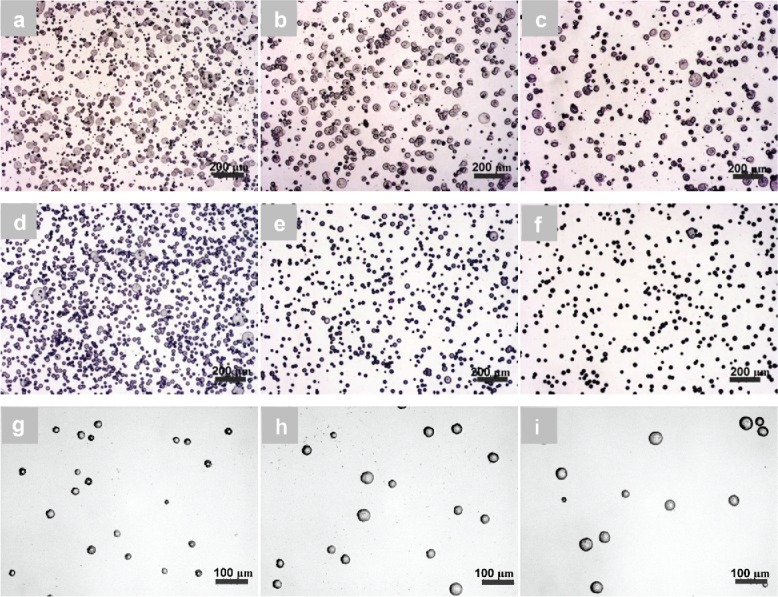
Effect of working distance on the morphology and size of PCL particles electrosprayed from PCL-2. (a) 1 wt.%−10 cm; (b) 1 wt.%−12.5 cm; (c) 1 wt.%−15 cm; (d) 5 wt.%−10 cm; (e) 5 wt.%−12.5 cm; (f) 5 wt.%−15 cm; (g) 7 wt.%−15 cm; (h) 7 wt.%−17.5 cm; and (i) 7 wt.%−20 cm.

Uniformly sized spherical particles were difficult to produce using a 1 wt.% PCL-2 solution, particularly when using a 10 cm working distance (Figure 5a). Increasing the working distance at this solution concentration improved the size uniformity and reduced the presence of larger particles (Figures 5b and c). There were significant changes (*p* < 0.05) in the mean particle diameters from 18.6 ± 7.6 μm, through 20.8 ± 5.7 μm to 27.6 ± 9.5 μm for a 1 wt.% PCL solution (Table 3). When 5 wt.% and 7 wt.% PCL-2 solutions were used, spherical PCL particles were produced at all three working distances (Figures 5d– i). At first, there was no significant change in diameter from 23.8 ± 6.8 μm to 24.3 ± 6.6 μm (*p* > 0.05) and then a significant increase (*p* < 0.05) to 26.3 ± 6.2 μm with an increasing working distance at a 5 wt.% concentration. In the case of 7 wt.% PCL solution, particle sizes increased significantly (*p* < 0.05) from 21.1 ± 3.0 μm to 28.0 ± 4.2 μm, and then increased less significantly (*p* > 0.05) to 30.0 ± 7.1 μm when the working distance was varied between 15 cm and 20 cm. Two-way ANOVA showed a significant relationship (*p* < 0.05) between the solution concentration and working distance. The histograms in Table 3 reveal the size distributions of PCL particles from 1 wt.%, 5 wt.%, and 7 wt.% PCL-2 solutions. Particles with diameters exceeding 40 μm were present for 1 wt.% and 5 wt.% PCL solutions. PCL particles electrosprayed from a 7 wt.% PCL solution had a relatively narrow diameter distribution, centralized around 25–30 μm and 30–35 μm for 15 cm and 17.5 cm working distances, respectively. For the particles produced using a working distance of 20 cm, the particle percentage in the range of 30–35 μm was similar to that in the range of 40–45 μm.

**Figure 6.**
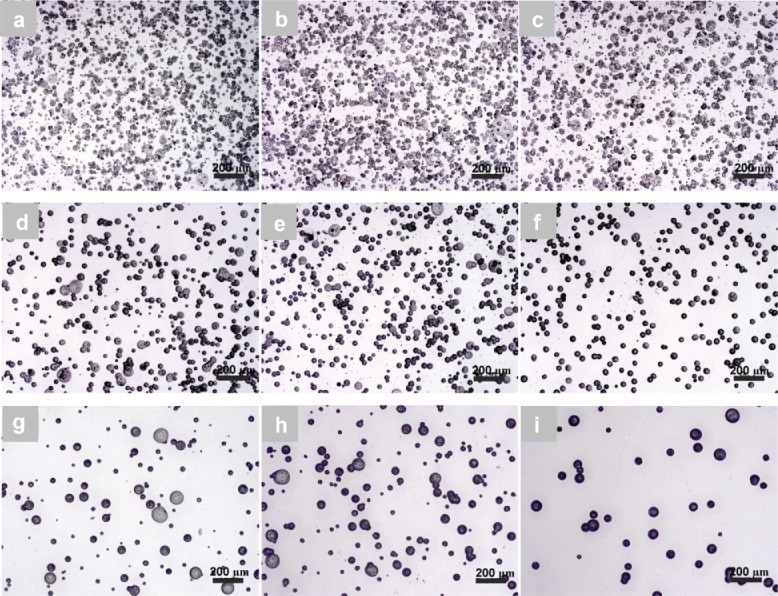
Effect of working distance on the morphology and size of PCL particles electrosprayed from PCL-3. (a) 1 wt.%−10 cm; (b) 1 wt.%−12.5 cm; (c) 1 wt.%−15 cm; (d) 5 wt.%−10 cm; (e) 5 wt.%−12.5 cm; (f) 5 wt.%−15 cm; (g) 9 wt.%−15 cm; (h) 9 wt.%−20 cm; (i) 9 wt.%−25 cm.

**Table 3.** Mean and standard deviations of PCL particle diameters and their distributions produced under different working distances.

Working distance *d_n_* (µm) Solution concentration	10 cm	12.5 cm	15 cm	Particles diameter distributions
1 wt.%	18.6 ± 7.6 μm	20.8 ± 5.7 μm	27.6 ± 9.5 μm	
5 wt.%	23.8 ± 6.8 μm	24.3 ± 6.6 μm	26.3 ± 6.2 μm	
7 wt.%^*^	21.1 ± 3.0 μm	28.0 ± 4.2 μm	30.0 ± 7.1 μm	

* For the 7 wt.% PCL solution, the working distance was 15 cm, 17.5 cm, and 20 cm. The relationship between sphere sizes and working distance/solution concentration was also plotted using *x-y-z* spatial coordinates. These plots were included as supplementary material.

For the PCL-3 solution with a 1 wt.% concentration, when the working distance was varied from 10 cm to 15 cm, it was observed that the cone-jet was not stable, resulting in PCL particles with polydisperse sizes (average diameters were ∼20 μm); no significant change (*p* > 0.05) in PCL particle average sizes was however observed, as shown in Figures 6a–c, indicating that the working distance was not as influential as for the 1 wt.% PCL-2 solution. When the PCL concentration was increased to 5 wt.%, the electrospraying jet became stable, resulting in less polydisperse particle sizes; the diameters of resultant particles however changed significantly (*p* < 0.05) from 30.0 ± 12.7 μm to 34.2 ± 12.8 μm with an increase in the working distance from 10 cm to 12.5 cm, but did not change significantly (35.8 ± 8.0 μm; *p* > 0.05) with an increase in the working distance to 15 cm (Figures 6d–f). When the PCL concentration was increased to 9 wt.%, there were noticeable increases (ANOVA, *p* < 0.05) in the mean particle diameter from 33.6 ± 17.3 µm, through 39.0 ± 18.0 μm, to 54.3 ± 15.8 µm when the working distance was increased from 17.5 cm to 25 cm (Figures 6g–i). Two-way ANOVA showed significant interactions (*p* < 0.05) between solution concentration and working distance. Table 4 shows the size distributions of electrosprayed particles from a PCL-3 solution with 1 wt.%, 5 wt.%, and 9 wt.% concentrations. There were no particles with diameters larger than 50 µm produced from 1 wt.% solution. Such PCL particles were present for a 5 wt.% solution and became more apparent (∼60 wt.%) in the case of a 9 wt.% solution. In addition, for the 5 wt.% and 9 wt.% solutions, with an increase in the working distance, PCL particles became more uniform. For example, around 40% of the particles had diameters in the range 35–40 µm at a working distance of 15 cm for a 5 wt.% solution but many fewer particles were in that range at both 10 cm and 12.5 cm working distances. For a 9 wt.% solution, 60% of the particles were larger than 50 µm when the working distance was increased to 25 cm from 15 cm. In this case, particles sizes also varied significantly.

**Table 4.** Mean and standard deviation of diameters of PCL-3 particles and their distributions produced under different working distances.

Working distance *d_n_* (µm) Solution concentration	10 cm	12.5 cm	15 cm	Particles diameter distributions
1 wt.%	18.9 ± 7.0 μm	20.1 ± 6.4 μm	20.6 ± 9.0 μm	
5 wt.%	30.0 ± 12.7 μm	34.2 ± 12.9 μm	35.8 ± 8.0 μm	
9 wt.%^*^	33.6 ± 17.3 μm	39.0 ± 18.0 μm	54.3 ± 15.8 μm	

* For the 9 wt.% PCL solution, the working distance was 15 cm, 20 cm, and 25 cm. The relationship between sphere sizes and working distance/solution concentration was also plotted using *x-y-z* spatial coordinates. These plots were included as supplementary material.

In general, larger PCL particles were generated with longer working distances, which was consistent with the trend observed for the electrospraying of poly lactide-co-glycolide (PLGA; Yao et al. 2008). However, it was also reported that the size of electrosprayed chitosan particles first increased and then decreased with an increasing working distance (Arya et al. 2009). A large working distance tended to produce particles with a spherical shape and a narrow size distribution, especially for a high concentration solution. This could be due to the fact that by increasing the working distance the polymer chains have sufficient time to diffuse within the droplet and thus reduce polydispersity (Bock et al. 2012). It was also found that the effect of working distance on particle sizes became less significant for PCL-2 solutions but more significant for PCL-3 solutions with increasing concentration. This could be caused by the higher level of molecular entanglement in PCL-2 than PCL-3, which restricts polymer chain diffusion.

### 3.4. Effect of flow rate

**Figure 7.**
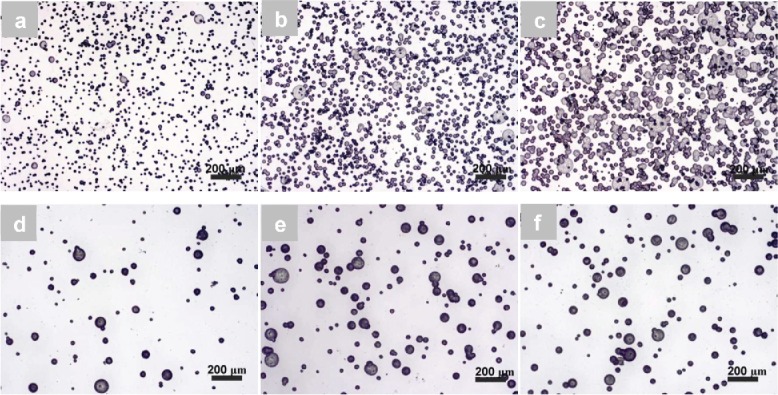
Effect of flow rate on the morphology and size of PCL particles. (a) PCL-2-5 wt.%− 0.3 mL h^−1^; (b) PCL-2-5 wt.%−0.6 mL h^−1^; (c) PCL-2-5 wt.%− 0.9 mL h^−1^; (d) PCL-3-9 wt.%−0.12 mL h^−1^; (e) PCL-3-9 wt.%−0.36 mL h^−1^; (f) PCL-3-9 wt.%− 0.6 mL h^−1^.

In the electrospraying/electrospinning process, a voltage–flow rate operating diagram is usually used to describe the jet behavior (Hohman et al. 2001a,b; Guarino et al. 2012). There is typically a stability window within which a steady cone-jet can form. It is well-known that the average sizes of electrosprayed particles increases with an increasing solution flow rate (Bock et al. 2011). In this study, 5 wt.% PCL-2 and 9 wt.% PCL-3 solutions were used to prepare particles under varying flow rates. The resultant particles from those solutions are shown in Figures 7a–f, respectively. It can be seen that particles from the 9 wt.% PCL-3 solution were much larger than those from 5 wt.% PCL-2. The mean diameter of PCL particles increased significantly (*p* < 0.05) from 18.0 ± 5.0 μm to 29.7 ± 9.6 μm (Table 5) and from 32.2 ± 15.0 μm to 41.1 ± 17.1 μm when the flow rate was varied from 5 μL min^−1^ to 15 μL min^−1^ and from 2 μL min^−1^ to 10 μL min^−1^, respectively. Interestingly, Bonferroni-corrected *post hoc* tests found that the former increase in flow rate resulted in a more significant change in particle size than the latter for both PCL-2 and PCL-3 solutions. This indicates that particle sizes could be more effectively controlled by the flow rate when it is varied from the lower limit of the stability window. Additionally, the size of particles from a 5 wt.% (PCL-2) solution was more uniform when a low flow rate was used (Figure 7a–c); the sizes of all particles produced from a 9 wt.% PCL-3 solution had a large variability with a changing flow rate (Figure 7d–f).

**Table 5.** Mean PCL particle diameter and standard deviation and their distributions produced under different flow rates.

Flow rate *d_n_* (µm) Solution concentration	0.3 mL h^−1^	0.6 mL h^−1^	0.9 mL h^−1^	Particle diameter distributions
5 wt.% (PCL-2)	18.0 ± 5.0 μm	23.8 ± 6.8 μm	29.7 ± 9.6 μm	
9 wt.%^*^ (PCL-3)	32.2 ± 15.0 μm	39.0 ± 17.9 μm	41.1 ± 17.1 μm	

* For the 9 wt.% PCL solution, the flow rate was 0.12 mL/h, 0.36 mL/h, and 0.6 mL/h.

The relationship between sphere sizes and flow rate/solution concentration was also plotted using *x-y-z* spatial coordinates. These plots were included as supplementary material.

### 3.5. Effect of particle collector

**Figure 8.**
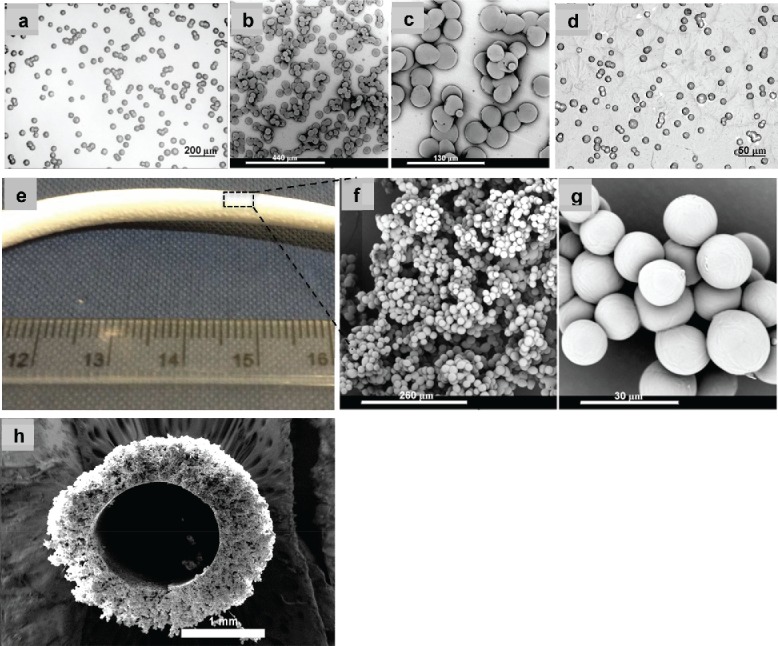
Optical and SEM micrographs of PCL particles electrosprayed from 9 wt.% PCL-2 solution on different collectors. (a) Glass slide; (b) aluminium foil; (c) close-up image of (b); (d) water bath; (e) digital image of tubular assembly of spheres on a copper wire; (f–h) SEM images with different magnifications of spheres in and cross-section of 3D tubular assembly.

In a typical electrospraying process, a solid, electrically conducting substrate (usually a grounded electrode) is used to collect particles. In a study (Wu and Clark 2007), a water bath was used to collect PCL particles electrosprayed from PCL/chloroform, resulting in a porous surface to the particles. Here four different types of collectors including a glass slide, aluminium foil, water bath, and a spring-shaped copper wire were employed to collect PCL particles from a 9 wt.% PCL-2 solution to study the deposited particles collected on these substrates. As shown in Figures 8a–c, PCL particles on the glass slide and aluminium foil tended to stick together to form aggregates, especially after an extended electrospraying time. Moreover, it was also observed that only a small amount of particles could reach the surface of the grounded plate; this was especially true for long working distances. The majority of particles were found to be carried by the air flow, and finally deposit on other surfaces in the fume cupboard. Therefore, the collection efficiency of PCL particles onto a glass slide or aluminium foil was low. Similar observations on electrosprayed particle fusion and loss during collection were also reported for the electrospraying of chitosan (Arya et al. 2009).

Various collection systems, for example, a water bath and an air jet flow, have been introduced into the electrospraying process to avoid particle aggregation and improve collection rates (Xie et al. 2006; Xu and Hanna 2006). In the present study, a water bath was also used to collect PCL particles. However, a film was formed on the surface of the water bath (Figure 8d), which hampered the dispersion of the particles. This was similar to a report on the deposition of electrospun PCL nanofibers in distilled water, where deposited material was found to float on the surface (Kostakova et al. 2014). In order to more efficiently capture the particles that are carried in the air flow of the fume hood, a grounded spring-shaped metal wire was placed in the spraying zone between the spinneret and the grounded plate. It was observed that the electrosprayed PCL particles were effectively attracted to and deposited on the grounded wire, which resulted in the formation of 3D tubular structure after an extended operation time (Figure 8e). The electrospraying of 3D microparticles was a one-step process, compared to other 3D microparticle fabrication methods (Nukavarapu et al. 2008; Yu et al. 2015). The SEM micrographs in Figures 8f and g indicate that the collected particles on the surface of the copper wire had a smooth surface and a uniform size. However, sphere packing tended to become denser from the outside to inside of the tubular structure, as shown in Figure 8h.

Most recently, a number of novel spinnerets for the mass production of core–shell structured polymeric spheres/fibers have been reported (Sharma et al. 2012; Megdi et al. 2013; Vysloužilová et al. 2014; Labbaf et al. 2014; Qin and Jiang 2014; Yan et al. 2015; Mahalingam et al. 2015). It can be envisaged that 3D core–shell/hollow microspheres will be formed when such a collecting system is introduced into these processes. In the future we will explore in detail these novel spinnerets to produce bulk structures with core–shell structured microfibers/spheres, which usually took a few hours to prepare for their application in diffusion MRI (Hubbard et al. 2015; McHugh et al. 2015).

## 4. Conclusions

In summary, electrospraying was demonstrated to be a versatile method for generating PCL particles with controllable morphology, size, and structure, which could be tuned with polymer molecular weight, solution concentration, applied voltage, flow rate, working distance, and collector type. It was observed that PCL molecular weight and solution concentration had a large effect on the formation and morphology of particles. Only microfibers were produced by electrospraying from PCL-1 (Mn = 70,000−90,000 g mol^−1^) with 0.5 wt.% to 10 wt.% concentration. Microparticles were generated from both PCL-2 (45,000 g mol^−1^) and PCL-3 (10,000 g mol^−1^) solutions with various concentrations, the latter having a wider range of possible concentrations. In order to avoid fiber formation, an increased polymer concentration must be coupled with a decreased molecular weight, ensuring electrospraying occurs in the semi-dilute entangled regime, which leads to the formation of spherical and homogenous particles. The morphology and size of the resultant particles were influenced to different extents by the applied voltage, working distance, and flow rate, among which the effect of the applied voltage was most significant. There was however a narrow range of the applied voltage allowing a more stable electrospraying process. PCL particles could not be collected in a water bath as efficiently as a grounded copper wire collecting substrate due to particle aggregation on the water surface. Furthermore, a 3D tubular assembly of smooth and spherical PCL microparticles could be formed on the copper wire and could be used as a tumor cell mimic.

## Supplementary Material

UAST_1234707_Supplementary_file.zip
