# Supplementary material for: Preparation and characterization of polycaprolactone microspheres by electrospraying
Source: Aerosol Sci Technol. 2016 Sep 13;50(11):1201–15. doi: 10.1080/02786826.2016.1234707 (PMC5111097; doi:10.1080/02786826.2016.1234707)
Supplement: UAST_1234707_Supplementary_file.zip [file uast_a_1234707_sm5872.zip › UAST_1234707_Supplementary file.docx]

**Supplemental Information**

Preparation and characterization of polycaprolactone microspheres by electrospraying

Feng-Lei Zhou ^1,2,^*, Penny L. Hubbard Cristinacce ^3^, Stephen J. Eichhorn ^4^ and Geoff J. M. Parker ^1,5^*

^1^Centre for Imaging Sciences, The University of Manchester, Manchester M13 9PT, United Kingdom, ^2^The School of Materials, The University of Manchester, Manchester M13 9PL, United Kingdom, ^3^School of Psychological Sciences, The University of Manchester, Manchester M13 9PT, United Kingdom, ^4^College of Engineering, Mathematics and Physical Sciences, North Park Road, University of Exeter, Exeter EX4 4QF, United Kingdom, ^5^Bioxydyn Limited, Rutherford House, Manchester Science Park, Pencroft Way, Manchester M15 6SZ, United Kingdom.

*Corresponding authors

Tel: +44 (0)161 275 5731; Fax: +44 (0)161 275 5145; Emails: geoff.parker@manchester.ac.uk and fenglei.zhou@manchester.ac.uk


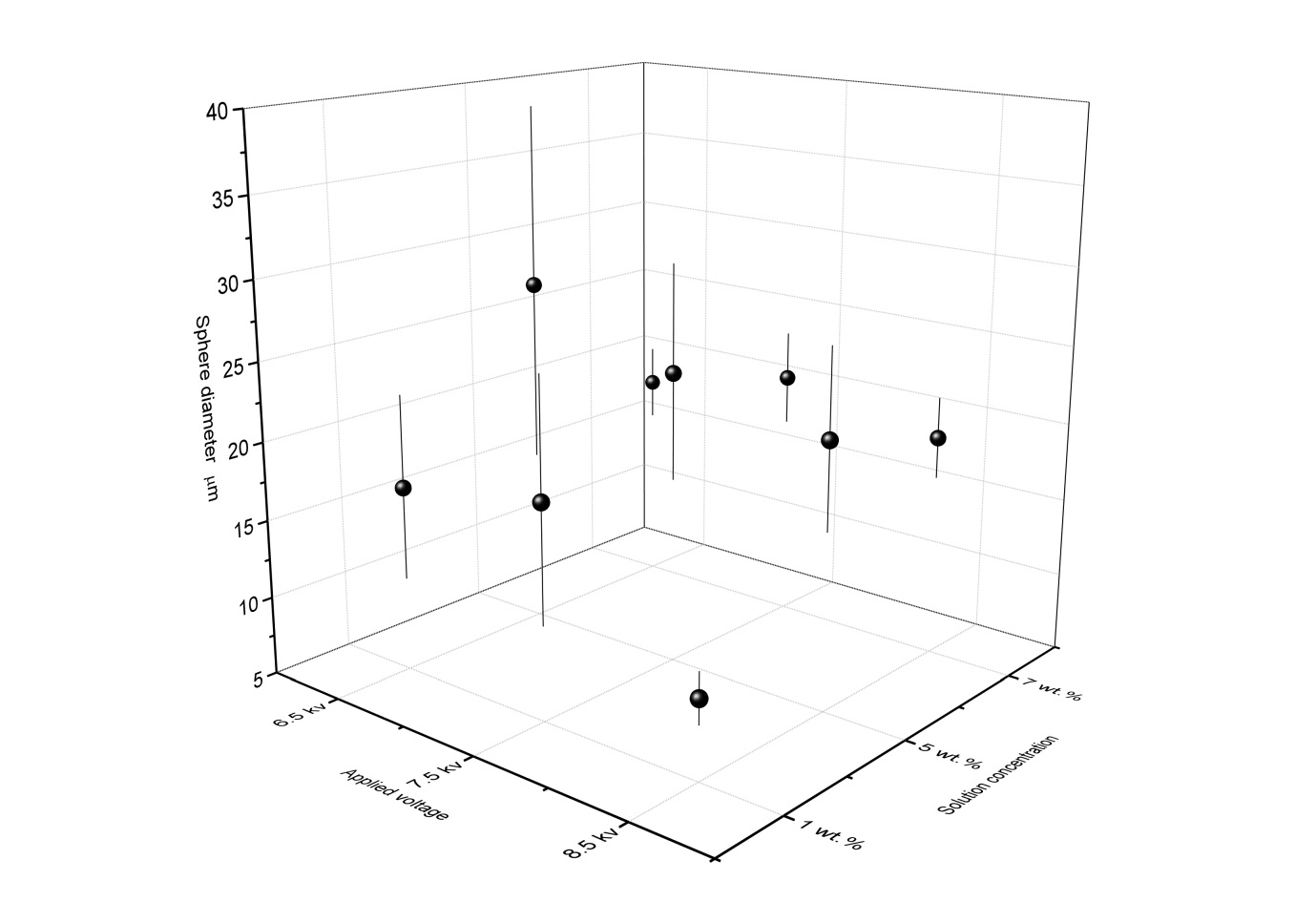


Figure S1. Mean and standard deviation of sphere diameters in Table 1.


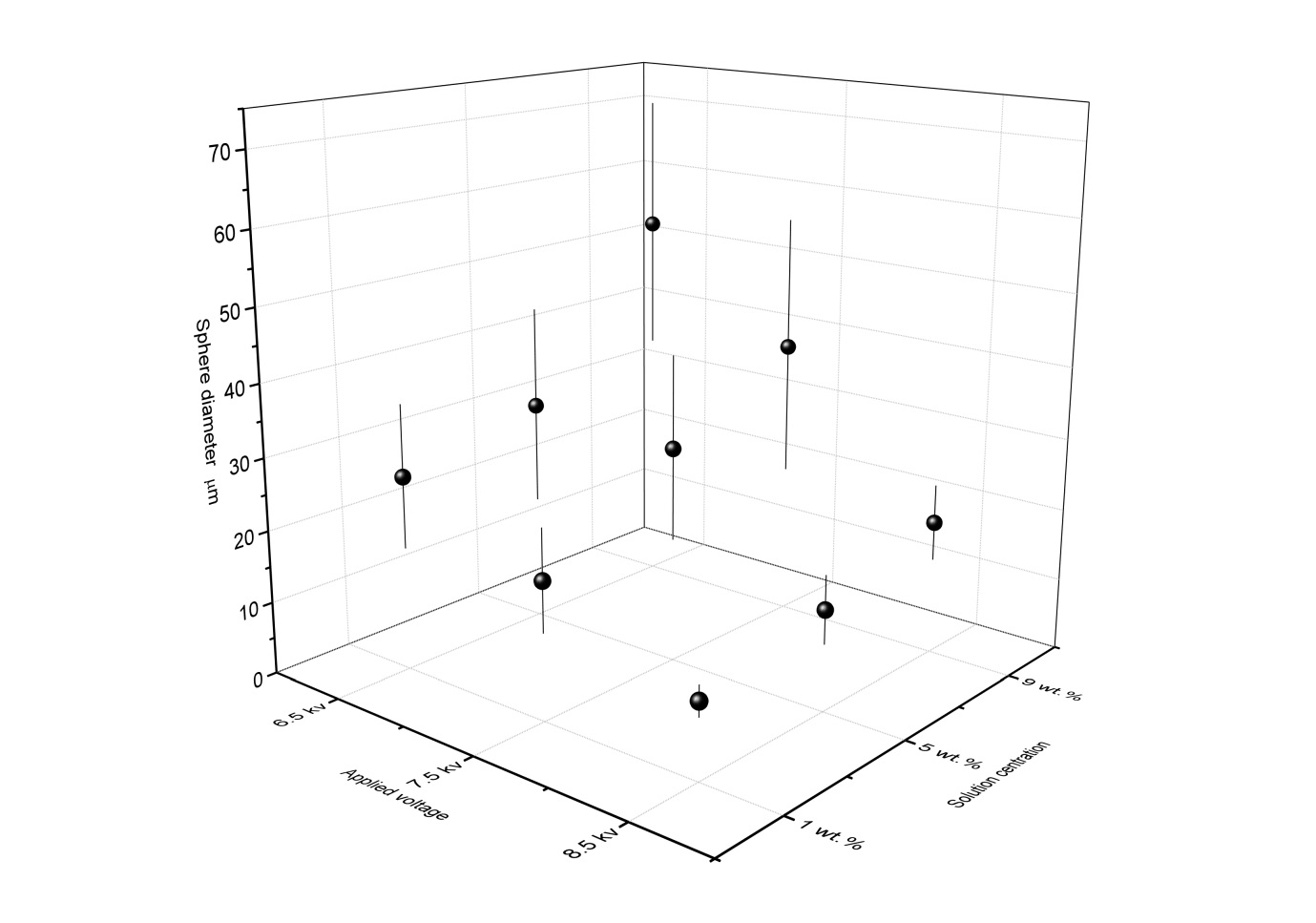


Figure S2. Mean and standard deviation of sphere diameters in Table 2.


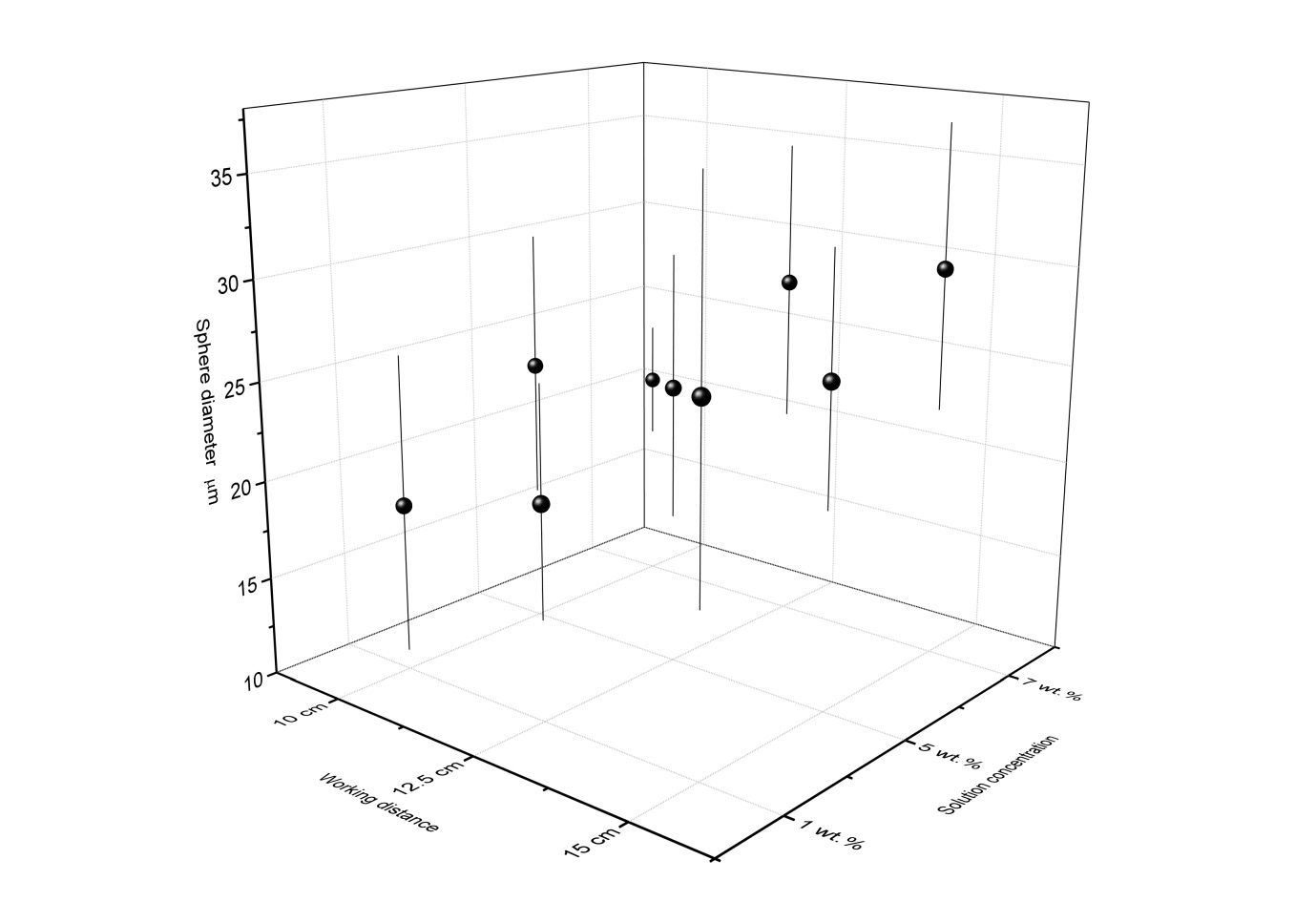


Figure S3. Mean and standard deviation of sphere diameters in Table 3.


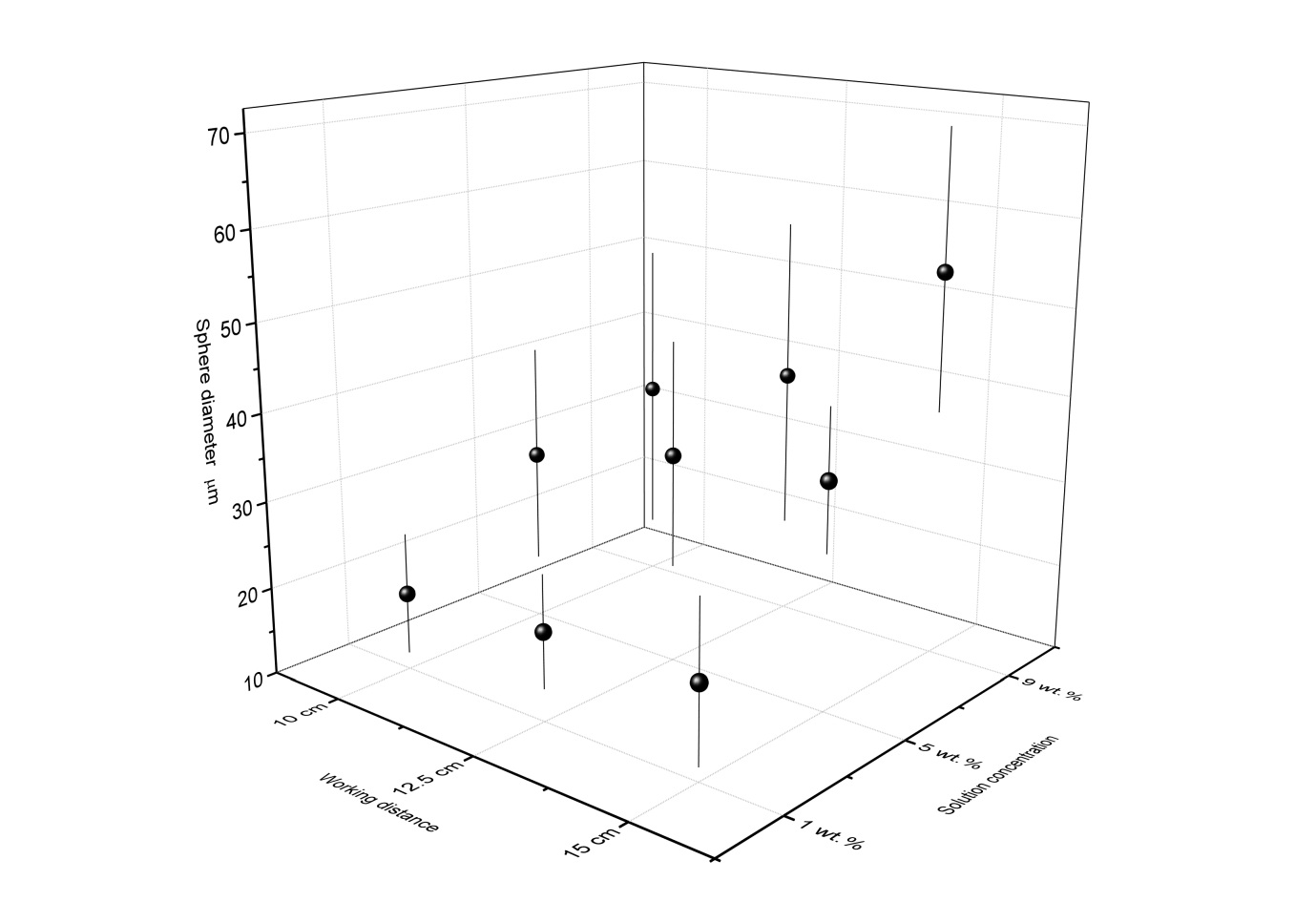


Figure S4. Mean and standard deviation of sphere diameters in Table 4.


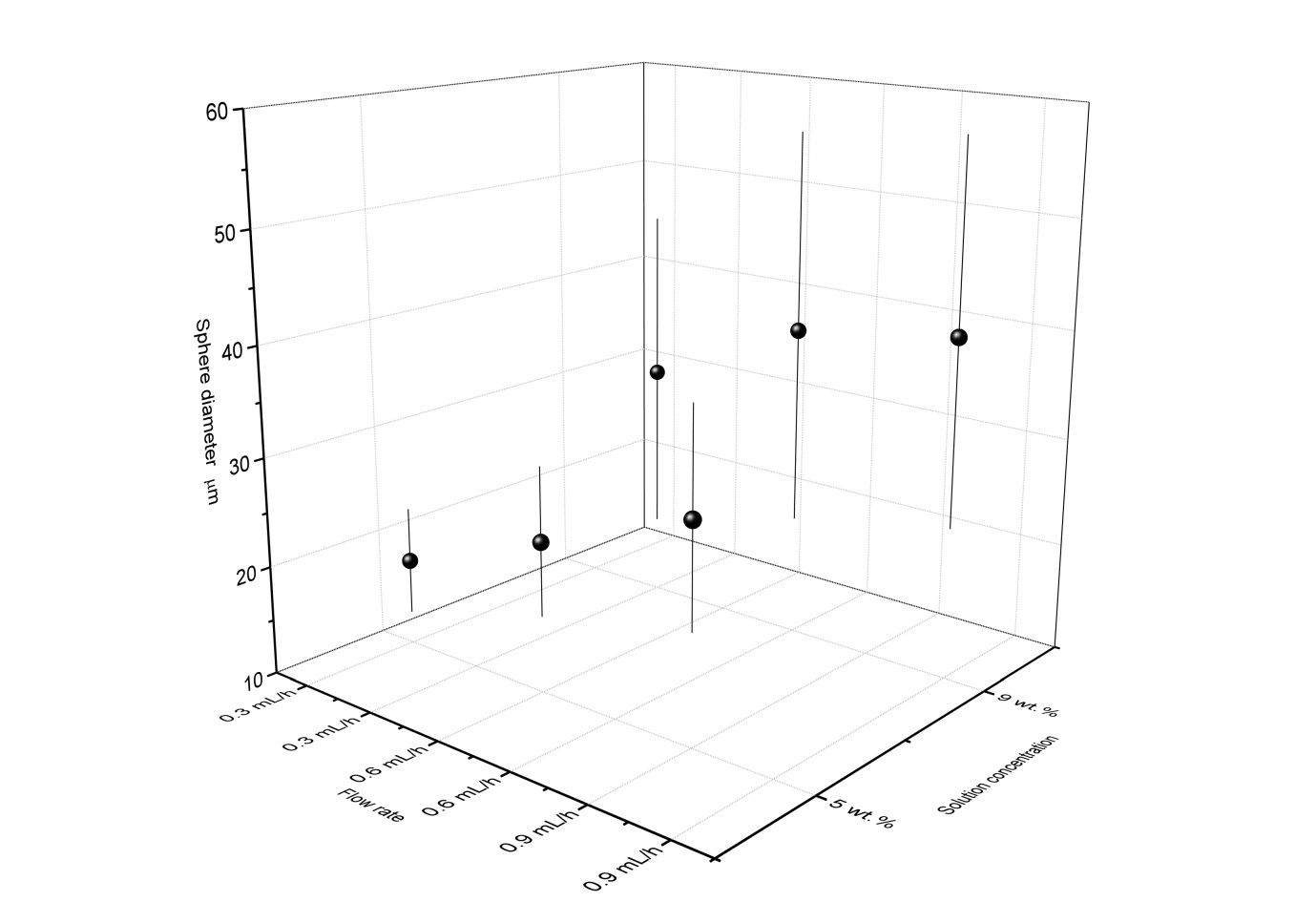


Figure S5. Mean and standard deviation of sphere diameters in Table 5.
